# Association of triglyceride glucose index and volumetric bone mineral density in T2DM: fat distribution as a potential indirect link

**DOI:** 10.3389/fcell.2026.1811476

**Published:** 2026-06-30

**Authors:** Bei Huang, Feng Xu, Jun-Jie Yang, Peng-Xin Wang, Zi-Fei Liu, Feng-Rong Liu, Hai-Yang Li, Ling-Qing Yuan, Jun Liu, Xiao Lin

**Affiliations:** 1 Department of Radiology, The Second Xiangya Hospital of Central South University, Changsha, China; 2 Department of Metabolism and Endocrinology, National Clinical Research Center for Metabolic Diseases, The Second Xiangya Hospital of Central South University, Changsha, China; 3 Department of Radiology, The Second Affiliated Hospital of Xinjiang Medical University, Ürümqi, China; 4 Department of Anesthesiology, The Xiangya Hospital of Central South University, Changsha, China; 5 Department of Radiology Quality Control Center in Hunan Province, Clinical Research Center for Medical Imaging in Hunan Province, Changsha, China

**Keywords:** bone mineral density, fat distribution, the triglyceride glucose index, the visceral-to-subcutaneous fat ratio, type 2 diabetes mellitus

## Abstract

**Background:**

Patients with type 2 diabetes mellitus (T2DM) generally present with insulin resistance, central obesity, and increased risk of fragility fracture. But the factors contributing to impaired bone health in T2DM are still unclear. This study investigated the association between the triglyceride glucose index (TyG), a surrogate marker of insulin resistance, and volumetric bone mineral density (vBMD) in T2DM, with a particular focus on the potential role of fat distribution.

**Methods:**

In this cross-sectional study, 189 patients with T2DM underwent quantitative computed tomography to assess lumbar vBMD and abdominal fat distribution, including visceral adipose tissue (VAT), subcutaneous adipose tissue (SAT), and the visceral-to-subcutaneous fat ratio (VSR). Participants were stratified by TyG tertiles. Spearman correlation analyses, multiple linear regression analyses, and mediation analyses were conducted to examine the associations among TyG, vBMD and fat distribution parameters.

**Results:**

Higher TyG correlated with a worse fat distribution pattern and lower vBMD. In the fully adjusted model, each unit increase in TyG was associated with a 10.420 mg/cm^3^ decrease in vBMD (β: 10.420, 95% CI: 14.476 to −6.364, *p* < 0.001). Mediation analyses demonstrated a statistically significant indirect effect of TyG on vBMD through both VAT and VSR, consistent with a potential intermediary role, with VSR accounting for the largest proportion of the association (32.76%).

**Conclusion:**

Elevated TyG is associated with lower vBMD in patients with T2DM, and this association is consistent with a possible indirect link through fat distribution, especially VSR. TyG may serve as a simple and cost-effective marker to identify impaired bone health in T2DM, while targeting unhealthy fat distribution may have potential value in preventing diabetic osteoporosis.

## 1 Introduction

Type 2 diabetes mellitus (T2DM) and its related bone complications represent a growing global health burden. T2DM is associated with abnormal bone metabolism, degraded bone microarchitecture, and increased bone fragility, which collectively elevate the risk of osteoporosis (OP) and fractures (Martiniakova et al., 2024). Insulin resistance (IR), defined as reduced sensitivity of peripheral tissues to insulin, is a crucial pathophysiological driver of T2DM (Reaven, 1988). Beyond its impact on glucose metabolism, IR negatively affects bone health through various mechanisms, including chronic inflammation, oxidative stress, and inhibition of osteoblast activity, thereby significantly influencing bone mineral density (BMD) (Jiao et al., 2015; Luo et al., 2025).

Recent research has shifted focus from overall obesity to the critical role of fat distribution in metabolic and bone health (Grune et al., 2025). Adipose tissue acts as an active endocrine organ, releasing a variety of adipokines and cytokines that directly link inflammation, bone remodeling, and metabolism (Lenchik et al., 2003; Hemat Jouy et al., 2024). A vicious cycle exists between IR and the pathological remodeling of fat distribution, through which IR may further compromise bone health. On the one hand, IR promotes the abnormal accumulation of visceral adipose tissue (VAT) (Lopes et al., 2016). As a metabolically active tissue, VAT secretes pro-inflammatory factors (e.g. IL-6 and TNF-α) and modulates adipokines (e.g. leptin and adiponectin), thereby inhibiting osteogenesis and promoting osteoclastic activity (Russell et al., 2010; Halade et al., 2011; Picó et al., 2022). On the other hand, subcutaneous adipose tissue (SAT) is considered to possess energy-buffering and anti-inflammatory effects, which potentially exert protective effects on bone health (Janssen, 2024). However, IR-related lipotoxicity can lead to SAT storage saturation, impairing its protective function (Pereira et al., 2022).

The homeostasis model assessment of insulin resistance (HOMA-IR) is widely used in epidemiological studies, yet it has several limitations. Beyond its reliance on single fasting measurements, its accuracy is affected by the lack of standardization across insulin assays, the influence of pulsatile insulin secretion, and limited use in patients with advanced β-cell dysfunction or those receiving exogenous insulin therapy. The hyperinsulinemic-euglycemic clamp (HIEC) is considered the gold standard for quantifying IR, but it is invasive, time-consuming, expensive and resource-intensive, making it not suitable for routine clinical practice (Park et al., 2021; Gastaldelli, 2022). In contrast, the triglyceride glucose index (TyG) has emerged as a simple and cost-effective surrogate marker that reflects both IR and glycolipid metabolism disorders. Notably, TyG has shown strong correlations with both HIEC and HOMA-IR, supporting its validity as a reliable surrogate marker of IR (Longo et al., 2025). Given the influence of IR on BMD, TyG has been extensively investigated for its relationship with BMD. However, evidence regarding this association remains inconclusive. Some studies report a nagetive association between TyG and BMD, while others suggest a protective association or even no significant link (Yousefiasl et al., 2025). The reliance on areal BMD (aBMD) measured by dual-energy X-ray absorptiometry (DXA), which is susceptible to artifacts from ectopic calcification or soft tissue thickening, may partly explain the conflicting results reported in the previous literature (Kim et al., 2021). To date, the relationship among TyG, BMD, and fat distribution has not been systematically investigated in the T2DM population, which typically characterized by IR, central obesity, and increased fragility fracture risk. Elucidating the underlying mechanisms in this population is crucial for accurately assessing their OP risks.

Within this context, quantitative computed tomography (QCT) presents distinct advantages. In a single scan, QCT can not only accurately measure volumetric BMD (vBMD), but also quantify multiple fat distribution parameters, including VAT and SAT. Given the independent effects of VAT and SAT on bone and metabolism, it may be inadequate to evaluate their overall detrimental impact by focusing solely on either fat distribution parameter. The visceral-to-subcutaneous fat ratio (VSR) provides a comprehensive perspective by quantifying the relative proportion of VAT and SAT, and may better reflect the impact of adverse fat distribution on health than VAT or SAT alone (Kaess et al., 2012; Sureka et al., 2023).

Therefore, this study aims to preliminarily explore the association between TyG and vBMD in patients with T2DM using QCT, with a particular focus on the potential roles of fat distribution in this relationship. By integrating fat quantity and distribution into the assessment framework, we seek to clarify whether TyG-related bone impairment is linked to specific adiposity patterns. This work is expected to provide new insights into metabolic–skeletal interactions and to inform the design of future larger-scale studies aimed at developing more precise, mechanism-based strategies for OP risk stratification and prevention in T2DM.

## 2 Materials and methods

### 2.1 Study design and participants

This single-center, cross-sectional study included patients with T2DM from the China Osteoporosis Risk-Stratified Cohort Research (CHOICE) in the Second Xiangya Hospital of Central South University between June 2024 and February 2025. T2DM was diagnosed based on the criteria established by the American Diabetes Association (ADA) (American Diabetes Association Professional Practice Committee, 2024). The inclusion criteria were as follow: (1) age ≥18 years; (2) confirmed diagnosis of T2DM; (3) agreement to undergo QCT examination. Patients were excluded if they met any of the following conditions: (1) other severe endocrine or autoimmune diseases known to affect bone metabolism; (2) long-term use of glucocorticoids or anti-osteoporosis medications; (3) severe hepatic or renal dysfunction (chronic kidney disease stage 4–5 or Child-Pugh class B/C liver disease); (4) lumbar deformities, internal fixation devices or other factors interfering with the accuracy of QCT measurement; (5) incomplete clinical data.

**FIGURE 1:**
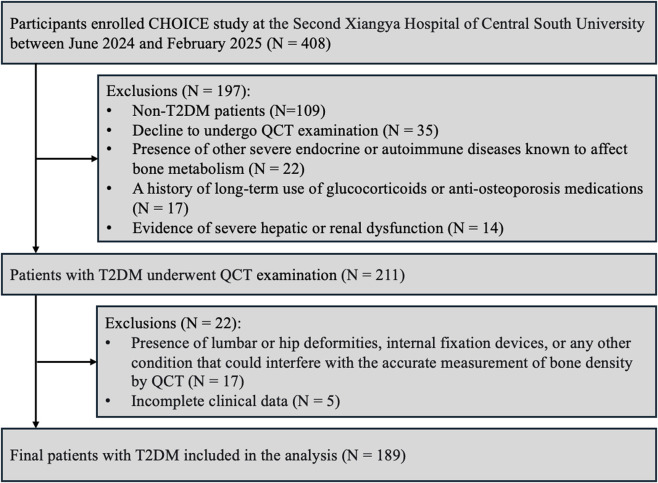
Flow chart of participant selection for the study. Abbreviations: CHOICE, the China Osteoporosis Risk-Stratified Cohort Research; T2DM, type 2 diabetes mellitus; QCT, quantitative computed tomography.

A total of 408 participants were enrolled in the CHOICE study. After strict screening according to the inclusion and exclusion criteria, 189 patients with T2DM were included in the final analysis, as shown by the flowchart (Figure 1). The study protocol was approved by the committee of the Second Xiangya Hospital of Central South University (approval number: 2022K037), and all procedures were conducted in accordance with the principles of the Declaration of Helsinki. Written informed consent was obtained from all participants.

### 2.2 Data collection

We collected demographic characteristics and medical histories of the study population, including age, sex, disease duration and medication use. Disease duration was defined as the interval in years between the first physician-confirmed diagnosis of T2DM and study enrollment. Medication use was recorded at enrollment based on current use status. Dyslipidemia medication use was defined as the use of any lipid-lowering medication at enrollment, and insulin therapy was defined as use of any insulin regimen at enrollment. Trained nurses conducted measurements of height, weight, and waist circumference (WC). Body mass index (BMI) was calculated as BMI = weight (kg)/height^2^ (m^2^).

Venous blood samples were obtained from all participants following a fasting period of ≥12 h for biochemical analysis. The measured parameters included fasting blood glucose, glycated hemoglobin (HbA1c), estimated glomerular filtration rate (eGFR), total cholesterol (TC), triglycerides (TG), high-density lipoprotein cholesterol (HDL-C), and low-density lipoprotein cholesterol (LDL-C). TyG was calculated using the formula: TyG = ln [fasting triglyceride (mg/dL) × fasting glucose (mg/dL)/2] (Alizargar et al., 2020).

### 2.3 Covariate selection

Covariates were selected based on their potential associations with both metabolic status and bone health, as well as their clinical relevance reported in previous literature (Conway et al., 2016; Kidney Disease: Improving Global Outcomes (KDIGO) CKD-MBD Update Work Group, 2011; Pan et al., 2024). Age and sex were included as basic demographic factors that are known to influence BMD. WC was included as an indicator of central adiposity, which was more closely aligned with the focus of present study on abdominal fat distribution and metabolic risk than BMI (Khanna et al., 2026). HbA1c was included to reflect long-term glycemic control (Mukherjee et al., 2024). eGFR was included to account for renal function, which may affect mineral metabolism and bone health (Evenepoel et al., 2023).

Disease duration was included as an indicator of cumulative diabetes exposure and disease progression (Sajjadi et al., 2024). Dyslipidemia medication and insulin therapy were included as treatment-related covariates because they may influence the metabolic background relevant to TyG. In particular, dyslipidemia medication may affect serum TG levels, which constitute a core component of the TyG (Bergmark et al., 2024), whereas insulin therapy directly affects fasting glucose and overall glycolipid metabolism, and may also reflect greater diabetes severity and treatment intensity in clinical practice (Fang et al., 2025). Insulin therapy was kept in the analysis because it was the antidiabetic drug variable that could be identified most reliably in this cohort and was considered the most clinically informative treatment-related indicator. In contrast, detailed information on non-insulin antidiabetic drug classes was not available for all patients.

### 2.4 Imaging examination

Participants were scanned in the supine position using a Siemens 96 × 2-slice dual-source CT scanner (Somatom Force, Siemens Healthineers, Germany). Prior to scanning, calibration was conducted using a QCT calibration phantom to ensure measurement accuracy. The scanning parameters were as follows: a tube voltage of 120 kV, automatic mAs, and a slice thickness of 1.0 mm. The scan range extended from the 11th thoracic vertebra to the third lumbar vertebra (T11-L3). The acquired images were transmitted to the Mindways QCT PRO system (Mindways Software Inc., Austin, TX, United States) for analysis.

### 2.5 vBMD and body compositions measurement

**FIGURE 2:**
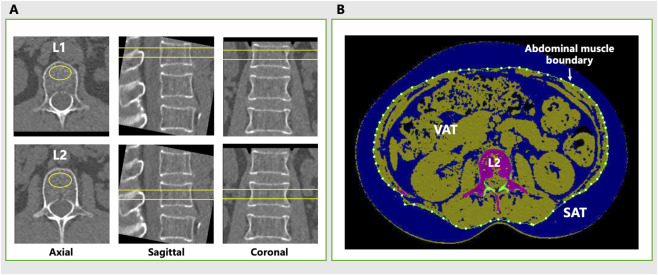
QCT-based assessment of lumbar vBMD and abdominal fat distribution. **(A)** Lumbar vBMD was measured by placing ROIs within the trabecular bone of the L1 and L2 vertebral bodies on axial, coronal, and sagittal images. **(B)** At the mid-L2 level, QCT PRO software automatically delineated the abdominal muscle boundary to differentiate SAT (between the muscle and skin) from VAT (within the abdominal cavity). Abbreviations: QCT, quantitative computed tomography; vBMD, volumetric bone mineral density; ROIs, regions of interest; SAT, subcutaneous adipose tissue; VAT, visceral adipose tissue.

On axial CT images, regions of interest (ROIs) were carefully positioned within the central areas of the L1 and L2 vertebral bodies to assess vBMD (Figure 2A). If the L1 or L2 level was unsuitable for measurement, an adjacent vertebral body was utilized as a substitute. To ensure accuracy, ROIs were positioned to avoid bone islands, sclerotic regions, basal vertebral veins, and cortical bone, while maximizing the ROI area. The mean lumbar vBMD was calculated as the average of the two measured vertebrae.

Fat distribution parameters were measured using the Tissue Composition module of QCT PRO software. A single axial image at the mid-vertebral level slice of L2 was utilized for analysis (Zeng et al., 2021). The software automatically delineated the outer boundary of the abdominal muscle. Body composition was differentiated via a threshold-based segmentation method, with fat defined as voxels exhibiting attenuation values between −195 and −45 Hounsfield units (HU) (Chen et al., 2020). The identified fat was categorized into VAT and SAT. SAT was defined as the adipose tissue located between the outer boundary of the abdominal muscle and the skin, while VAT encompassed all intra-abdominal adipose tissue areas (Figure 2B). Manual corrections were performed as necessary when automatic delineation was inaccurate.

### 2.6 Statistical analysis

Continuous variables were expressed as mean ± SD if normally distributed, or median (Q1–Q3) if skewed. Categorical variables were presented as frequency (percentage). Baseline characteristics were compared across TyG tertiles using ANOVA/Kruskal–Wallis tests for continuous variables and chi-square test for categorical variables. Spearman correlation assessed associations between key continuous variables. Multiple linear regression examined the relationships among TyG, fat distribution and vBMD across three models: Model 1 was unadjusted, Model 2 adjusted for age, sex, and WC, Model 3 was additionally adjusted for eGFR, HbA1c, disease duration, dyslipidemia medication and insulin therapy. Restricted cubic spline (RCS) regression with 3 knots (10th, 50th, and 90th percentiles) was used to assess potential non-linear relationship between TyG and vBMD after adjusting variables in Model 3. Sex-stratified analyses were conducted to examine whether the association between TyG and vBMD differed by sex. Mediation analyses were conducted using the bootstrap resampling (5,000 samples) to evaluate whether VAT, SAT, and VSR were associated with the link between TyG and vBMD. Because this study has a cross-sectional design, the results reflect statistical mediation consistent with a potential intermediary role, but no causal inference can be drawn. All analyses were performed using R software (Version 4.5.2), with two-sided p < 0.05 considered significant.

## 3 Results

### 3.1 Baseline characteristics

Table 1 presents baseline characteristics of 189 participants with T2DM, stratified by TyG tertiles. The cohort had a mean age of 56.81 ± 11.60 years, including 47.62% males and 52.38% females. As the TyG levels increased, participants showed significantly higher weight, BMI, and WC, along with a more adverse lipid profile, characterized by elevated TG, TC, and LDL-C levels, and reduced HDL-C levels (all *p* < 0.01). Fat distribution parameters analysis revealed a unique pattern: both VAT and VSR increased significantly across TyG tertiles (both *p* < 0.001). SAT showed a decreasing trend in the highest tertile, although the between-group difference did not reach statistical significance. vBMD demonstrated consistent decreasing trend with rising TyG levels (*p* < 0.001). Additionally, the use of dyslipidemia medication differed significantly among groups (*p* = 0.012). No significant differences were observed in age, sex, height, disease duration, HbA1c, eGFR, or insulin therapy.

**TABLE 1:** Baseline characteristics of study participants.

Variables^a^	Total	TyG tertiles			P value^b^
		T1 (7.14–8.74)	T2 (8.75–9.63)	T3 (9.63–12.88)	
N	189	63	63	63	​
Age (years)	56.81 ± 11.60	55.73 ± 12.48	58.90 ± 10.54	55.81 ± 11.60	0.216
Sex (N, %)	​	​	​	​	0.641
Male	90 (47.62%)	32 (50.79%)	27 (42.86%)	31 (49.21%)	​
Female	99 (52.38%)	31 (49.21%)	36 (57.14%)	32 (50.79%)	​
Height (cm)	160.67 ± 8.46	160.77 ± 8.25	159.56 ± 7.88	161.70 ± 9.20	0.364
Weight (kg)	62.08 ± 12.75	58.33 ± 10.96	61.66 ± 11.16	66.26 ± 14.71	**0.002**
BMI (kg/m^2^)	23.93 ± 3.71	22.49 ± 3.28	24.10 ± 3.33	25.19 ± 4.01	**<0.001**
WC (cm)	85.87 ± 11.59	81.80 ± 10.42	87.02 ± 10.86	88.78 ± 12.41	**0.002**
Disease duration^c^ (years)	10.60 ± 9.11	10.82 ± 9.33	10.75 ± 9.24	10.24 ± 8.87	0.929
HbA1c (%)	8.78 ± 2.75	8.65 ± 2.83	9.11 ± 2.63	8.58 ± 2.81	0.510
eGFR (ml/min/1.73m2)	88.50 ± 26.41	88.71 ± 26.03	85.85 ± 23.18	90.94 ± 29.77	0.558
TG (mmol/L)	1.42 (0.94, 2.15)	0.98 (0.73, 1.16)	1.50 (1.25, 1.94)	2.74 (1.75, 4.23)	**<0.001**
TC (mmol/L)	4.46 ± 1.18	4.16 ± 1.13	4.22 ± 1.14	4.99 ± 1.11	**<0.001**
HDL-C (mmol/L)	1.08 (0.87, 1.35)	1.20 (1.04, 1.43)	1.07 (0.86, 1.42)	0.93 (0.82, 1.18)	**<0.001**
LDL-C (mmol/L)	2.63 ± 0.98	2.46 ± 0.98	2.48 ± 0.92	2.94 ± 0.97	**0.007**
VAT (cm^2^)	151.92 ± 69.80	112.18 ± 50.09	166.53 ± 70.11	177.06 ± 69.88	**<0.001**
SAT (cm^2^)	107.40 ± 59.58	114.26 ± 63.44	112.15 ± 60.27	95.81 ± 53.84	0.164
VSR	1.74 ± 1.00	1.24 ± 0.88	1.78 ± 0.82	2.21 ± 1.04	**<0.001**
vBMD (mg/cm^3^)	108.08 ± 38.72	124.97 ± 36.43	103.49 ± 32.52	95.79 ± 41.18	**<0.001**
Dyslipidemia medication^c^ (N, %)	133 (70.37%)	36 (57.14%)	51 (80.95%)	46 (73.02%)	**0.012**
Insulin therapy^c^ (N, %)	122 (64.55%)	46 (73.02%)	36 (57.14%)	40 (63.49%)	0.173

^a^ Normally distributed continuous variables were expressed as mean ± standard deviation; Nonnormally distributed continuous variables were expressed as median (Q1-Q3); Categorical variables were expressed as frequency and percentage.

^b^ Significant P values are shown in bold.

^c^ Disease duration was defined as the interval between the first diagnosis of T2DM and study enrollment. Dyslipidemia medication and insulin therapy were recorded based on use at enrollment and coded as binary variables (yes/no).

TyG, the triglyceride glucose index; BMI, body mass index; WC, waist circumference; HbA1c, glycated hemoglobin; eGFR, estimated glomerular filtration rate; TG, triglycerides; TC, total cholesterol; HDL-C, high-density lipoprotein cholesterol; LDL-C, low-density lipoprotein cholesterol; VAT, visceral adipose tissue; SAT, subcutaneous adipose tissue; VSR, the visceral-to-subcutaneous fat ratio; vBMD, volumetric bone mineral density.

### 3.2 Correlations among key variables

Spearman correlation analyses were performed to preliminarily evaluate the relationships among TyG, vBMD, fat distribution parameters, and potential confounders (Supplementary Figure S1). TyG was significantly negatively correlated with vBMD (r = −0.392, *p* < 0.001). Notably, TyG was positively correlated with VAT (r = 0.427, *p* < 0.001) and more strongly with VSR (r = 0.505, *p* < 0.001), while showing a weak negative correlation with SAT (r = −0.149, *p* = 0.040). Both VAT and VSR were significantly negative correlated with vBMD (r = −0.395 and −0.372, respectively; both *p* < 0.001). Surprisingly, BMI was also negatively correlated with vBMD (r = −0.258, *p* < 0.01).

### 3.3 Association between TyG and vBMD

Multiple linear regression analyses were performed to examine whether TyG was independently associated with vBMD in patients with T2DM. As shown in Table 2, higher TyG levels were consistently associated with lower vBMD across all models. In the fully adjusted model (Model 3), each unit increase in TyG was associated with a decrease of 10.420 mg/cm^3^ in vBMD (β: 10.420, 95% CI: 14.476 to −6.364, *p* < 0.001).

**TABLE 2:** Associations between TyG and vBMD.

Variables	Model 1		Model 2		Model 3	
	β (95%CI)	P value	β (95%CI)	P value	β (95%CI)	P value
TyG (continuous)	−12.092 (−16.995, −7.189)	**<0.001**	−10.370 (−14.353, −6.388)	**<0.001**	−10.420 (−14.476, −6.364)	**<0.001**
TyG (tertiles)						
T1 (7.14–8.74)	Reference	-	Reference	-	Reference	-
T2 (8.75–9.63)	−21.477 (−34.441, −8.514)	**0.001**	−12.112 (−22.714, −1.511)	**0.025**	−11.834 (−22.889, −0.779)	**0.036**
T3 (9.63–12.88)	−29.177 (−42.140, −16.213)	**<0.001**	−24.695 (−35.371, −14.019)	**<0.001**	−24.408 (−35.273, −13.543)	**<0.001**
P For trend	**<0.001**	​	**<0.001**	​	**<0.001**	​

Bold values indicate statistical significance.

TyG, the triglyceride glucose index; vBMD, volumetric bone mineral density; WC, waist circumference; HbA1c, glycated hemoglobin; eGFR, estimated glomerular filtration rate.

Model 1: no covariates were adjusted.

Model 2: age, sex and WC were adjusted.

Model 3: age, sex, WC, eGFR, HbA1c, disease duration, dyslipidemia medication and insulin therapy were adjusted.

When TyG was analyzed by tertiles, both the middle (β: 11.834, 95% CI: 22.889 to −0.779, *p* = 0.036) and highest tertiles (β: 24.408, 95% CI: 35.273 to −13.543, *p* < 0.001) exhibited lower vBMD compared with the lowest tertile after adjusting for Model 3, with a significant linear trend across tertiles (p for trend <0.001). In addition, the RCS further suggested an approximately linear relationship between TyG and vBMD (p for nonlinear = 0.185; Supplementary Figure S2). Given well-known differences in fat distribution and bone loss patterns between men and women, we performed sex-stratified analyses to capture potential sex-specific associations (Kim et al., 2025). The results showed that TyG was negatively associated with vBMD in both males (β: 8.620, 95% CI: 15.096 to −2.144, *p* = 0.010) and females (β: 11.648, 95% CI: 17.184 to −6.113, *p* < 0.001), but the interaction between sex and TyG was not statistically significant (*p* = 0.657; Supplementary Figure S3). These results indicate that higher TyG is linked to decreased vBMD in patients with T2DM, independent of metabolic and clinical confounders.

### 3.4 Associations of fat distribution with TyG and vBMD

To explore the potential role of fat distribution in the association between TyG and vBMD, we examined the relationships of fat distribution parameters with both TyG and vBMD. As shown in Table 3, TyG was positively associated with VAT (β: 16.881, 95% CI: 9.332 to 24.431, *p* < 0.001) and VSR (β: 0.360, 95% CI: 0.241 to 0.478, *p* < 0.001) in fully adjusted models (Model 3), while it was inversely associated with SAT only after covariate adjustment (β: −11.875, 95% CI: −19.003 to −4.746, *p* = 0.001).

**TABLE 3:** Associations between TyG and fat distribution parameters.

Outcomes	Model 1			Model 2			Model 3		
	β (95%CI)	Std β	P value	β (95%CI)	Std β	P value	β (95%CI)	Std β	P value
VAT	25.061 (16.404, −33.719)	0.385	**<0.001**	17.379 (9.940, 24.817)	0.267	**<0.001**	16.881 (9.332, 24.431)	0.260	**<0.001**
SAT	−6.705 (−14.655, −1.245)	−0.121	0.098	−12.317 (−19.602, −5.033)	−0.222	**0.001**	−11.875 (−19.003, −4.746)	−0.214	**0.001**
VSR	0.372 (0.249, 0.495)	0.401	**<0.001**	0.380 (0.257, 0.503)	0.410	**<0.001**	0.360 (0.241, 0.478)	0.387	**<0.001**

Bold values indicate statistical significance.

TyG, the triglyceride glucose index; VAT, visceral adipose tissue; SAT, subcutaneous adipose tissue; VSR, the visceral-to-subcutaneous fat ratio; TyG, the triglyceride glucose index; WC, waist circumference; HbA1c, glycated hemoglobin; eGFR, estimated glomerular filtration rate.

Model 1: no covariates were adjusted.

Model 2: age, sex and WC were adjusted.

Model 3: age, sex, WC, eGFR, HbA1c, disease duration, dyslipidemia medication and insulin therapy were adjusted.

We next evaluated the correlation between fat distribution parameters and vBMD. As presented in Table 4, both VAT (β: −0.148, 95% CI: −0.225 to −0.071, *p* < 0.001) and VSR (β: −12.735, 95% CI: −17.282 to −8.188, *p* < 0.001) were negatively associated with vBMD after adjusting for Model 3, whereas SAT showed a positive association only after covariate adjustment (β: 0.117, 95% CI: 0.032–0.202, *p* = 0.008). Notably, among all fat distribution parameters, VSR demonstrated the strongest association with both TyG (Standardized β: 0.387) and vBMD (Std β: 0.328).

**TABLE 4:** Associations between fat distribution parameters and vBMD.

Variables	Model 1			Model 2			Model 3		
	β (95%CI)	Std β	P value	β (95%CI)	Std β	P value	β (95%CI)	Std β	P value
VAT	−0.201 (−0.275, −0.126)	−0.362	**<0.001**	−0.145 (−0.221, −0.069)	−0.262	**<0.001**	−0.148 (−0.225, −0.071)	−0.267	**<0.001**
SAT	0.003 (−0.091, 0.096)	0.004	0.955	0.110 (0.029, 0.191)	0.170	**0.008**	0.117 (0.032, 0.202)	0.180	**0.008**
VSR	−10.203 (−15.613, −4.794)	−0.263	**<0.001**	−11.715 (−15.974, −7.456)	−0.301	**<0.001**	−12.735 (−17.282, −8.188)	−0.328	**<0.001**

Bold values indicate statistical significance.

vBMD, volumetric bone mineral density; VAT, visceral adipose tissue; SAT, subcutaneous adipose tissue; VSR, the visceral-to-subcutaneous fat ratio; TyG, the triglyceride glucose index; WC, waist circumference; HbA1c, glycated hemoglobin; eGFR, estimated glomerular filtration rate.

Model 1: no covariates were adjusted.

Model 2: age, sex and WC were adjusted.

Model 3: age, sex, WC, eGFR, HbA1c, disease duration, dyslipidemia medication and insulin therapy were adjusted.

Overall, these results support that fat distribution may serve as an important intermediate link connecting TyG-related metabolic disturbances with reduced vBMD in T2DM.

### 3.5 Fat distribution as a potential indirect link between TyG and vBMD

**FIGURE 3:**
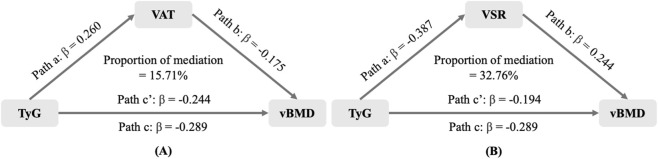
Statistical mediation models of VAT and VSR in the association between TyG and vBMD. **(A)** VAT and **(B)** VSR as variables associated with the TyG–vBMD relationship. Path a represents the association between TyG and the mediator. Path b represents the association between the mediator and vBMD after adjustment for TyG. Path c represents the total effect of TyG on vBMD, whereas path c′ represents the direct effect of TyG on vBMD after inclusion of the mediator. The product of paths a and b (a × b) represents the indirect effect. Both VAT and VSR showed statistically significant indirect associations, consistent with a potential intermediary role, with VSR accounting for a larger proportion of the TyG–vBMD relationship. All models were adjusted for age, sex, WC, eGFR, HbA1c, disease duration, dyslipidemia medication, and insulin therapy. Abbreviations: VAT, visceral adipose tissue; VSR, the visceral-to-subcutaneous fat ratio; TyG, the triglyceride glucose index; vBMD, volumetric bone mineral density; WC, waist circumference; eGFR, estimated glomerular filtration rate; HbA1c, glycated hemoglobin.

Mediation analyses were performed to explore whether fat distribution is associated with the link between TyG and vBMD in patients with T2DM (Table 5; Figure 3). After adjusting for age, sex, WC, eGFR, HbA1c, disease duration, dyslipidemia medication and insulin therapy, both VAT and VSR showed statistically significant indirect associations, consistent with a potential intermediary role. Notably, the proportion of VSR (32.76%) was markedly higher than that of VAT (15.71%). In contrast, SAT was not significantly associated with the TyG–vBMD relationship. These findings suggest that the association between TyG and vBMD in patients with T2DM may be partly explained by fat distribution, with VSR accounting for the largest proportion of the association.

**TABLE 5:** Mediation analysis of fat distribution parameters in the association between TyG and vBMD.

Variables	Path a		Path b		Path c		Path c’		Indirect effect		Proportion (%)
	β (95% CI)	P value	β (95% CI)	P value	β (95% CI)	P value	β (95% CI)	P value	β (95% CI)	P value	
VAT	0.260 (0.144, 0.375)	**<0.001**	−0.175 (−0.315, −0.035)	**0.015**	−0.289 (−0.401, −0.177)	**<0.001**	−0.244 (−0.359, −0.128)	**<0.001**	−0.045 (−0.091, −0.010)	**0.008**	15.71%
SAT	−0.214 (−0.341, −0.086)	**0.001**	0.109 (−0.018, 0.237)	0.095	−0.289 (−0.401, −0.177)	**<0.001**	−0.265 (−0.380, −0.151)	**<0.001**	−0.023 (−0.056, 0.005)	0.109	8.10%
VSR	0.387 (0.260, 0.514)	**<0.001**	−0.244 (−0.368, −0.120)	**<0.001**	−0.289 (−0.401, −0.177)	**<0.001**	−0.194 (−0.312, −0.076)	**0.002**	−0.095 (−0.179, −0.035)	**<0.001**	32.76%

Bold values indicate statistical significance.

VAT, visceral adipose tissue; SAT, subcutaneous adipose tissue; VSR, the visceral-to-subcutaneous fat ratio; TyG, the triglyceride glucose index; vBMD, volumetric bone mineral density; WC, waist circumference; HbA1c, glycated hemoglobin; eGFR, estimated glomerular filtration rate.

Covariates: age, sex, WC, eGFR, HbA1c, disease duration, dyslipidemia medication and insulin therapy.

Path a: from TyG to the mediator; Path b: from the mediator to vBMD; Path c’: from TyG to vBMD with mediator adjustment; and Path c: from TyG to vBMD without mediator adjustment.

## 4 Discussion

This study investigated the relationships among TyG, QCT-derived vBMD, and fat distribution in patients with T2DM. According to the study results, higher TyG levels were independently associated with lower vBMD and with an unfavorable fat distribution pattern, characterized by increased VAT, a higher VSR, and reduced SAT. Both VAT and VSR were inversely associated with vBMD. Mediation analysis further revealed that fat distribution was associated with the TyG–vBMD relationship, consistent with a potential intermediary role, with VSR accounting for a larger proportion than individual fat compartments such as VAT or SAT.

Previous DXA-based studies investigating the relationship between TyG and BMD have yielded inconsistent results. For example, Gu et al. found no significant association between TyG and BMD or OP in postmenopausal women with T2DM (Gu et al., 2023). Korpe et al. identified a negative correlation between TyG and lumbar BMD in non-diabetic postmenopausal women (Korpe et al., 2025). A cross-sectional study linked higher TyG levels to low bone mass and higher risk of OP (Zhuo et al., 2023). A systematic review suggested that such heterogeneity might be due to differences in study populations, diabetes status, BMI, and other confounding factors (Yousefiasl et al., 2025). Methodologically, these discrepancies may partly due to the limitations of DXA: ectopic calcification and central obesity–related soft tissue thickening, both common in patients with T2DM, can lead to pseudo-elevation of aBMD, masking the actual bone loss (Xu et al., 2019; Kim et al., 2021).

In contrast, QCT measurement of vBMD effectively distinguishes bone from calcification and minimizes soft tissue interference, thus providing a more accurate estimate of BMD (Xu et al., 2019). This methodological advantage may explain our ability to detect a clear negative association between TyG and vBMD, which might be obscured in DXA-based studies, thereby enhancing the reliability of our findings. Our findings underscore the clinical value of QCT for assessing skeletal status in patients with T2DM and challenge the common view that BMD is typically normal or elevated in T2DM (Ma et al., 2012). In addition, QCT simultaneously and precisely quantified fat distribution in a single scan, facilitating an integrated assessment of bone and fat phenotypes. This comprehensive approach highlights the utility of the QCT single imaging mode for evaluating metabolic bone disease risk without increasing the dose of radiation.

Mechanistically, the negative correlation between TyG and vBMD likely reflects the cumulative adverse effects of IR and related glycolipid metabolic disturbances on bone. TyG integrates information on fasting TC and blood glucose, captures potential IR and metabolic dysfunction states. In addition to decreasing insulin’s anabolic effects on bone and altering osteoclast activity, high TyG metabolic environment may also promote bone loss through oxidative stress, inflammation, and lipotoxicity (Jiao et al., 2015; Hu et al., 2019; Luo et al., 2025). Since TyG is obtained from routine laboratory tests, it may be useful in primary healthcare settings and large-scale epidemiological surveys as a simple and low-cost screening tool for bone health risks in patients with T2DM, and may support earlier risk stratification in clinical practice.

In this study, VAT was positively associated with TyG and negatively associated with vBMD, accounting for 15.71% of the TyG–vBMD relationship. Sharma et al. confirmed VAT as an independent negative predictor of bone metabolism markers (Sharma et al., 2020). IR promotes VAT accumulation, while VAT, as an active endocrine tissue, impairs bone metabolism through multiple mechanisms (Russell et al., 2010). On the one hand, VAT expansion leads to increased secretion of pro-inflammatory mediators such as TNF-α, IL-1β, and IL-6. These cytokines promote osteoclast differentiation and upregulate receptor activator of nuclear factor kappa-B ligand (RANKL) expression while suppressing osteoblast activity, shifting the balance of bone remodeling toward resorption (Forte et al., 2023). Halade et al. observed in diet-induced obese mice that VAT accumulation accompanied by elevated levels of pro-inflammatory mediators resulted in enhanced osteoclast formation and bone resorption, along with suppressed osteoblast development, ultimately reducing trabecular bone volume (Halade et al., 2011). On the other hand, dysregulated adipokines also contribute to bone remodeling. Adiponectin can alter the RANKL/OPG ratio in the bone marrow microenvironment, promoting osteoclastogenesis and inhibiting osteoblast activity (Yang et al., 2019); while leptin not only inhibits osteoclast formation through modulation of the RANKL/OPG axis, but also indirectly affects bone remodeling via hypothalamic regulation of energy balance (Picó et al., 2022; Stefanakis et al., 2024). Taken together, reducing VAT accumulation, combined with effective blood glucose control, could be considered a potential intervention target for preserving bone health in T2DM.

SAT exhibited a less consistent role. Although SAT was associated with both lower TyG and higher vBMD after multivariate adjustment, it did not show a statistically significant indirect association with the TyG–vBMD relationship. This possibly is related to the functional changes of SAT in the metabolic environment of T2DM. Under physiological conditions, SAT serves as an energy buffer and exerts anti-inflammatory effects, with its secretion of adiponectin contributing to anti-inflammatory actions and enhanced systemic insulin sensitivity (Booth et al., 2018; Zaidi et al., 2022; Janssen, 2024). However, in states of severe IR, the storage capacity of SAT may be impaired, leading to ectopic fat deposition and functional alterations (Santoro et al., 2021; Pereira et al., 2022; Janssen, 2024). In our cohort, the observed decreasing trend of SAT in the highest TyG tertile is consistent with reduced SAT storage under severe IR. These observations align with previous reports indicating SAT dysfunction and inflammatory activation in IR states (Mashayekhi et al., 2024). Therefore, while SAT may retain some protective effects, its contribution to the TyG–vBMD relationship appears limited in this cohort. Further studies are needed to validate how SAT function influences bone health in T2DM.

A key finding of this study is that VSR demonstrated the strongest association with both TyG and vBMD and accounted for the largest proportion of the TyG–vBMD relationship (32.76%). Due to the coexistence of VAT-related harmful effects and SAT dysfunction in T2DM, evaluating individual fat compartments may make it difficult to comprehensively assess their collective impact. VSR reflects the balance between metabolically harmful VAT and potentially protective SAT. A high VSR indicates a predominance of VAT over SAT, corresponding to a shift toward greater pro-inflammatory signaling and adipokine imbalance, cumulatively impairing osteoblast function and promoting osteoclast activity. Therefore, compared with single fat compartments, VSR may serve as a superior indicator for detecting the effects of fat distribution on bone remodeling.

Recently, VSR has received increasing attention in the assessment of metabolic diseases. Multiple studies have pointed out that VSR outperformed individual fat distribution parameters in predicting metabolic syndrome, non-alcoholic fatty liver disease, and other cardiometabolic events (Kaess et al., 2012; Sureka et al., 2023; Emamat et al., 2024). Our findings suggest that the overall pattern of fat distribution rather than the absolute amount of single fat, may be an important factor associated with IR-related bone impairment in T2DM. These findings highlight the limitations of traditional indices such as BMI and the clinical significance of fat distribution assessment. This suggests that promoting a healthier fat distribution pattern, rather than simple weight loss, may better protect bone health. This is consistent with the current emphasis on “fat distribution remodeling,” whereby reducing VAT and improving overall fat distribution through lifestyle and weight-management strategies may have additional value for preserving bone health in T2DM.

We also observed a negative correlation between BMI and vBMD, which contrasts with the view of obesity’s protective mechanical effect on bone (Shapses and Sukumar, 2012). Emerging evidence suggests a more complex relationship between obesity and skeletal health. For instance, Li et al. reported an inverted U-shaped relationship between BMI and lumbar BMD, suggesting that excessive weight may have a negative impact on the spine (Li, 2022). Moreover, the spine appears particularly sensitive to hormonal and endocrine changes (Naessén et al., 2006). In patients with T2DM, metabolic disorders centered on IR not only promote adverse visceral fat distribution, but may also exacerbate lumbar bone loss. The adverse metabolic and endocrine effects related to obesity, especially unhealthy fat distribution, may therefore overturn or reverse the mechanical benefits of weight on bone. The negative correlation between vBMD, TyG, BMI, and poor fat distribution parameters together indicates that visceral-dominant obesity worsens bone damage in T2DM, which provides potential insights for clinical risk stratification and individualized intervention.

To our knowledge, this is the first study to simultaneously investigate the relationships among TyG, vBMD, and fat distribution using QCT in patients with T2DM. The application of mediation analysis quantified the contributions of different fat compartments and indicated that VSR accounted for the largest proportion in the TyG–vBMD relationship, providing a more comprehensive perspective on potential links between metabolic alterations and bone impairment in T2DM. Our findings suggest that T2DM patients with both higher TyG and VSR levels may represent a subgroup at increased risk for low vBMD. While the exact thresholds still need validation in prospective studies, combining TyG and VSR measurements could form a practical risk-stratification framework for early bone health monitoring in T2DM. For example, in an endocrinology or diabetes clinic setting, TyG could be calculated from routine lab tests for initial screening, and patients with elevated TyG could then undergo abdominal QCT to assess VSR. Those with higher VSR could be considered for closer monitoring or further evaluation. This approach provides clinicians with concrete guidance to identify high-risk individuals and implement stratified surveillance, supporting early and proactive management of bone health in patients with T2DM.

This study has several limitations. Firstly, owing to the cross-sectional design of this study, the causal relationships among TyG, vBMD, and fat distribution inferred from the mediation analysis should be interpreted with caution. Secondly, the single-center design and relatively small sample size limit the generalizability of the findings. Thirdly, this cohort did not systematically collect information on specific non-insulin glucose-lowering medications, including sodium-glucose cotransporter 2 inhibitors (SGLT2i) and glucagon-like peptide-1 receptor agonists (GLP-1RAs). Evidence suggests that SGLT2i may reduce BMD, while GLP-1RAs are generally considered to have neutral or slightly positive effects on BMD (Lu et al., 2015; Jensen et al., 2024; Haarhaus, 2026). As these two drug classes are known to have opposing effects on bone health and their exact usage prevalence in our cohort is unknown, the net magnitude and direction of this residual confounding cannot be reliably quantified, and therefore unmeasured confounding related to these drugs cannot be fully ruled out (Egger et al., 2016; Daniilopoulou et al., 2022). Fourthly, fat assessment was confined to the abdomen and did not include other fat depots, and the influence of unmeasured factors such as diet, exercise levels, and vitamin D status cannot be completely ruled out. Lastly, QCT entails exposure to ionizing radiation, which restricts its use in all clinical contexts. Future large-scale prospective cohort studies and interventional trials are needed to validate the temporal and causal associations and to explore whether optimizing fat distribution can improve vBMD. Future studies should also consider the possible effects on bone health of non-insulin glucose-lowering medications. Mechanistic investigations should focus on specific adipokines and inflammatory factors to elucidate underlying biological pathways. Additionally, other fat depots and sex-specific differences should also be warranted.

## 5 Conclusion

This cross-sectional study provides preliminary evidence of independent associations among TyG, fat distribution, and QCT-derived vBMD in patients with T2DM. Notably, VSR accounted for the largest proportion of the TyG–vBMD relationship, consistent with a potential intermediary role of overall fat distribution rather than individual fat compartments. These findings suggest that TyG may serve as a simple, cost-effective tool for identifying OP risk in T2DM, and highlight the potential value of fat management for bone health.

## Data Availability

The raw data supporting the conclusions of this article will be made available by the authors, without undue reservation.
