# Supplementary material for: Association of triglyceride glucose index and volumetric bone mineral density in T2DM: fat distribution as a potential indirect link
Source: Front Cell Dev Biol. 2026 Jun 30;14:1811476. doi: 10.3389/fcell.2026.1811476 (PMC13364863; doi:10.3389/fcell.2026.1811476)
Supplement: Supplementary file 1 [file DataSheet1.docx]

**Supplemental Files**

**Supplement Figure 1. Correlations among key variables.** Circle color indicates the direction of the correlation, and circle size indicates the absolute value of Spearman’s rho. Abbreviations: vBMD, volumetric bone mineral density; TyG, the triglyceride glucose index; VAT, visceral adipose tissue; SAT, subcutaneous adipose tissue; VSR, the visceral-to-subcutaneous fat ratio; BMI, body mass index; WC, waist circumference; HbA1c, glycated hemoglobin; eGFR, estimated glomerular filtration rate.

**Supplement Figure 2. Restricted cubic spline analysis of the association between TyG and vBMD.** The x-axis represents TyG, and the y-axis represents the predicted difference in vBMD (ΔvBMD, mg/cm³) relative to the reference value at TyG P50 = 9.140. The solid line shows the estimated association, and the shaded area indicates the 95% confidence interval. The restricted cubic spline model was adjusted for age, sex, WC, eGFR, HbA1c, disease duration, dyslipidemia drug, and insulin therapy. The analysis showed an approximately linear inverse association between TyG and vBMD after multivariable adjustment. Abbreviations: TyG, the triglyceride glucose index; vBMD, volumetric bone mineral density; WC, waist circumference; HbA1c, glycated hemoglobin; eGFR, estimated glomerular filtration rate.

**Supplement Figure 3. Sex-stratified association between TyG and predicted vBMD in patients with T2DM.** Predicted vBMD by TyG in males and females after adjustment for age, WC, HbA1c, eGFR, disease duration, dyslipidemia medication, and insulin therapy. Sex-stratified analyses showed significant negative associations in both sexes, with a non-significant TyG × sex interaction (P = 0.657). Abbreviations: vBMD, volumetric bone mineral density; TyG, the triglyceride glucose index; WC, waist circumference; HbA1c, glycated hemoglobin; eGFR, estimated glomerular filtration rate.

**
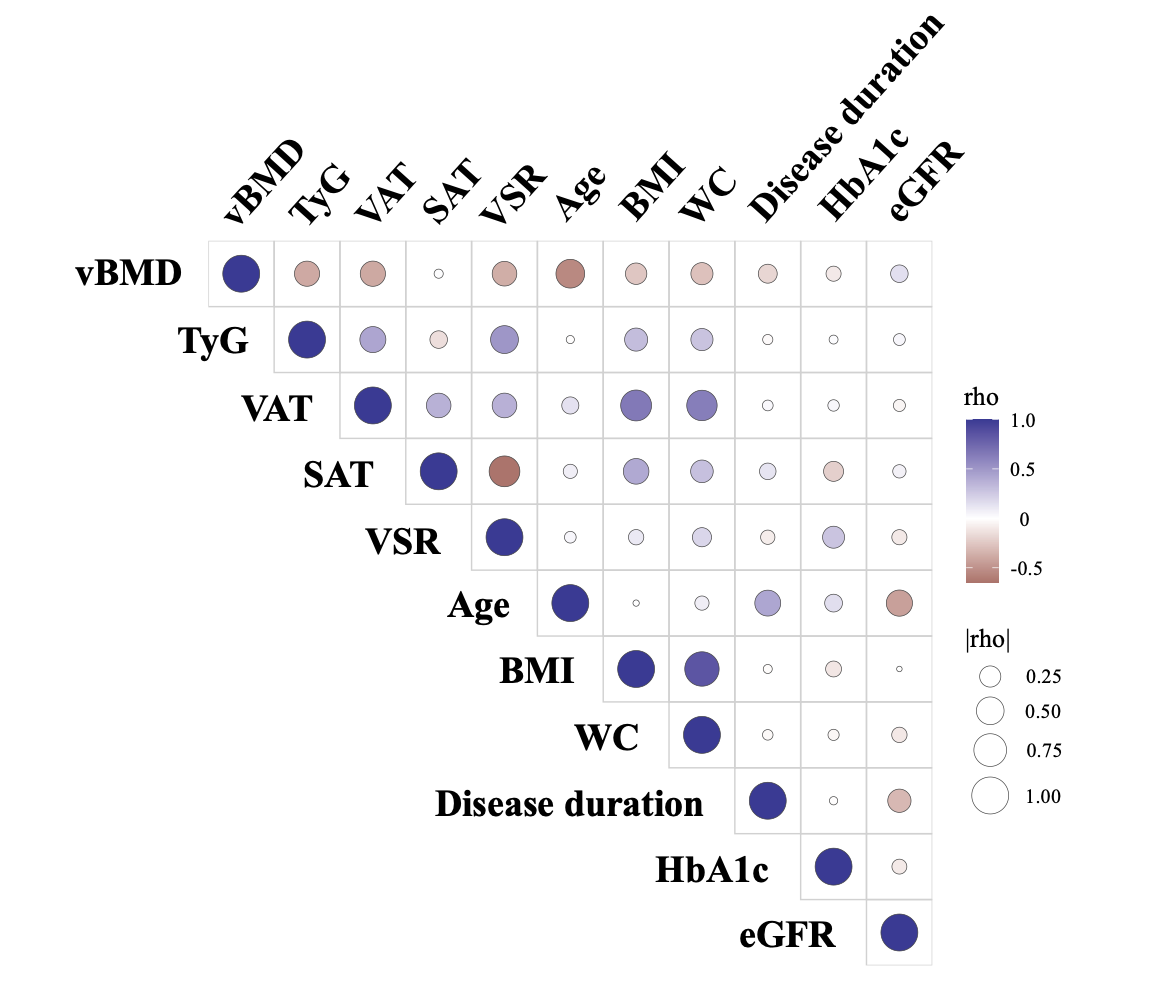
Supplement Figure 1**

**
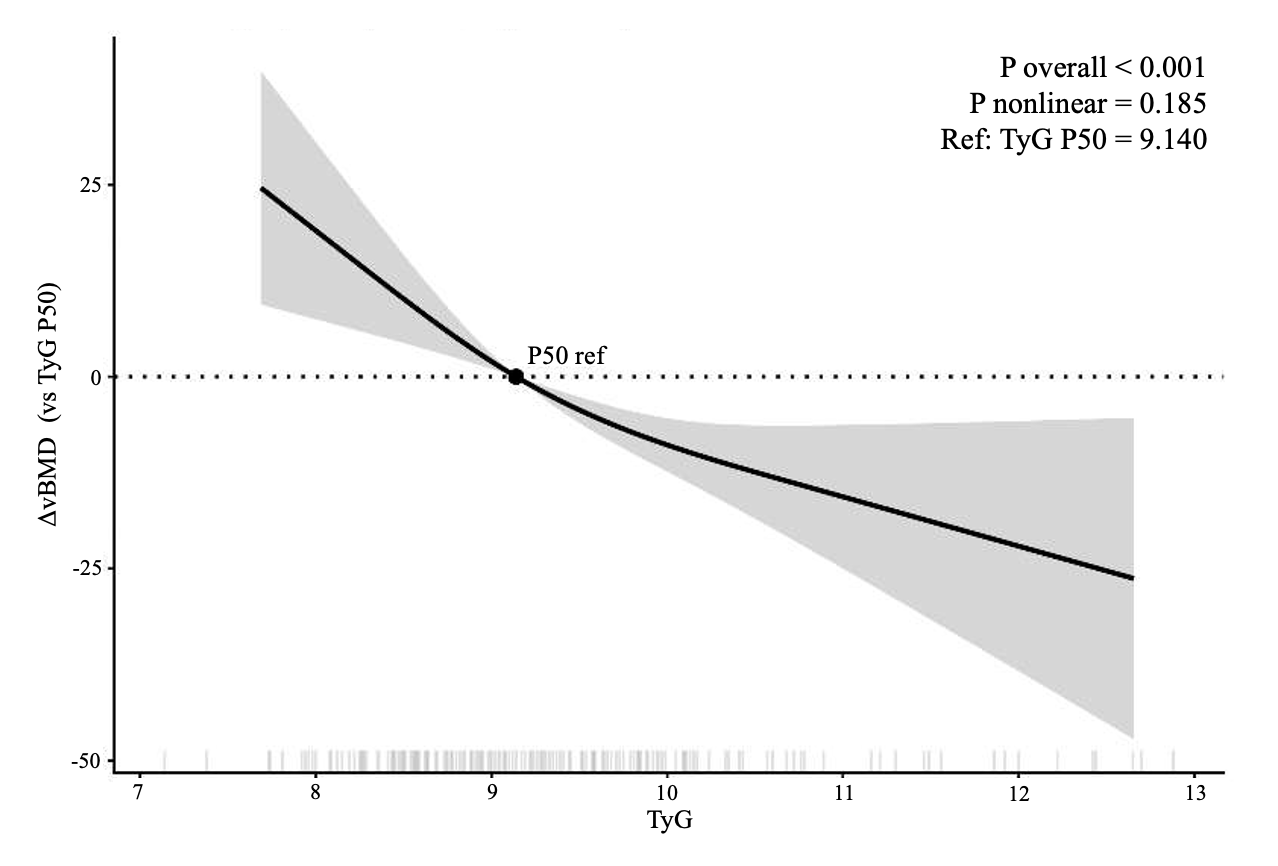
**

**Supplement Figure 2**

**
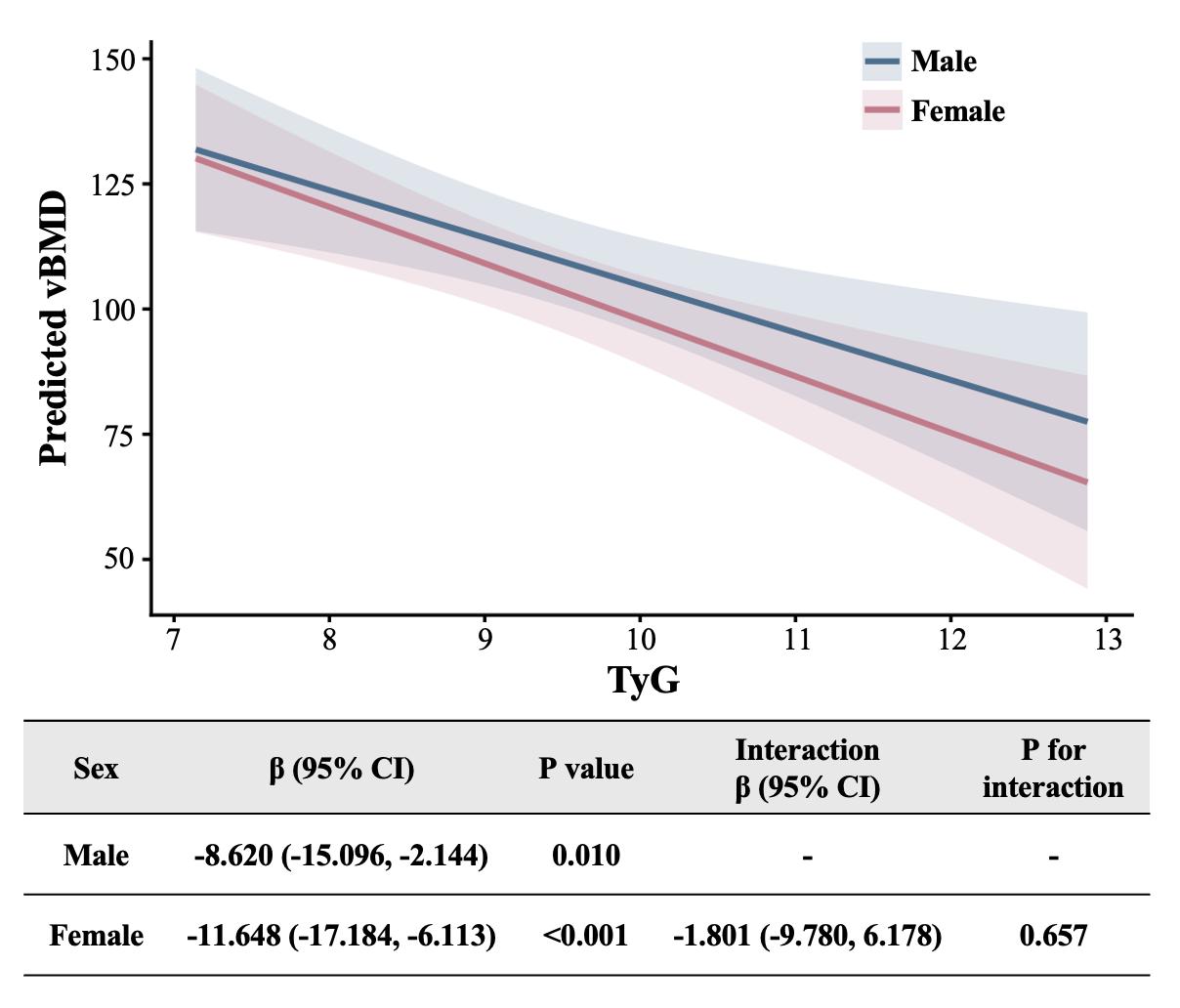
Supplement Figure 3**
